# Crystal structures of 2-meth­oxy-5-nitro­aniline *N*-alkyl derivatives support color-center creation by a dipole-stacking mechanism

**DOI:** 10.1107/S205698902600681X

**Published:** 2026-07-10

**Authors:** Jonathan Filley, Ethan J. Crace

**Affiliations:** a Oligometrics, Inc., 2510 47th Street, Suite 208, Boulder, CO 80301, USA; b Analytical Resources Core, Colorado State University, Fort Collins, CO 80523, USA; Vienna University of Technology, Austria

**Keywords:** crystal structure, color center, charge transfer, π-stacking

## Abstract

Two *N*-alkyl derivatives of 2-meth­oxy-5-nitro­aniline, namely, 2-meth­oxy-*N*-(4-methyl­benz­yl)-5-nitro­aniline (C_15_H_16_N_2_O_3_, orange crystals) and 2-meth­oxy-5-nitro-*N*-(tri­phenyl­meth­yl)aniline ethanol quatersolvate (C_26_H_22_N_2_O_3_·0.25C_2_H_5_OH, yellow crystals), have been prepared and analyzed by single-crystal X-ray diffraction to determine if these compounds display the same face-to-face π-stacking arrangement as observed in the un-alkyl­ated parent compound. The tri­phenyl­methyl derivative crystallizes with 0.25 ethanol mol­ecules, the contribution of which has been modeled with a solvent mask due to a high degree of disorder. The UV-Vis spectra show that the benzyl derivative displays concentration-dependent shifts in the visible portion of the spectrum, shifting to longer wavelengths with higher concentration, which is attributed to aggregation in solution. This behavior is similar to that observed for the parent compound. In contrast, the tri­phenyl­methyl derivative shows an attenuated concentration dependence on visible absorption, which does not display as great a red shift, and therefore represents a lesser tendency for aggregation in solution. The crystal packing for these compounds is distinguishing, with the benzyl derivative π-stacking in a face-to-face manner with dipole moments oriented anti-parallel, while the sterically demanding tri­phenyl­methyl group prevents direct π-stacking for this compound. The packing observed for these compounds supports the hypothesis that an inter­molecular charge-transfer process is responsible for the orange color of crystals of the benzyl derivative. It is concluded that sterically undemanding groups attached at the amino group could deliver materials which mimic the color center formation hypothesized for the parent compound. These types of structural changes may have useful applications in the field of organic semiconductors.

## Abstract 2

A striking color change from orange to yellow is observed in crystals of two *N*-alkyl derivatives of 2-meth­oxy-5-nitro­aniline. The orange compound has a sterically unencumbered 4-methyl­benzyl group and the yellow compound the more sterically demanding tri­phenyl­methyl group. The crystal structures are used to rationalize a charge-transfer color center in the former compound, which is less favorable in the latter.

## 1. Chemical context

**Figure 1:**
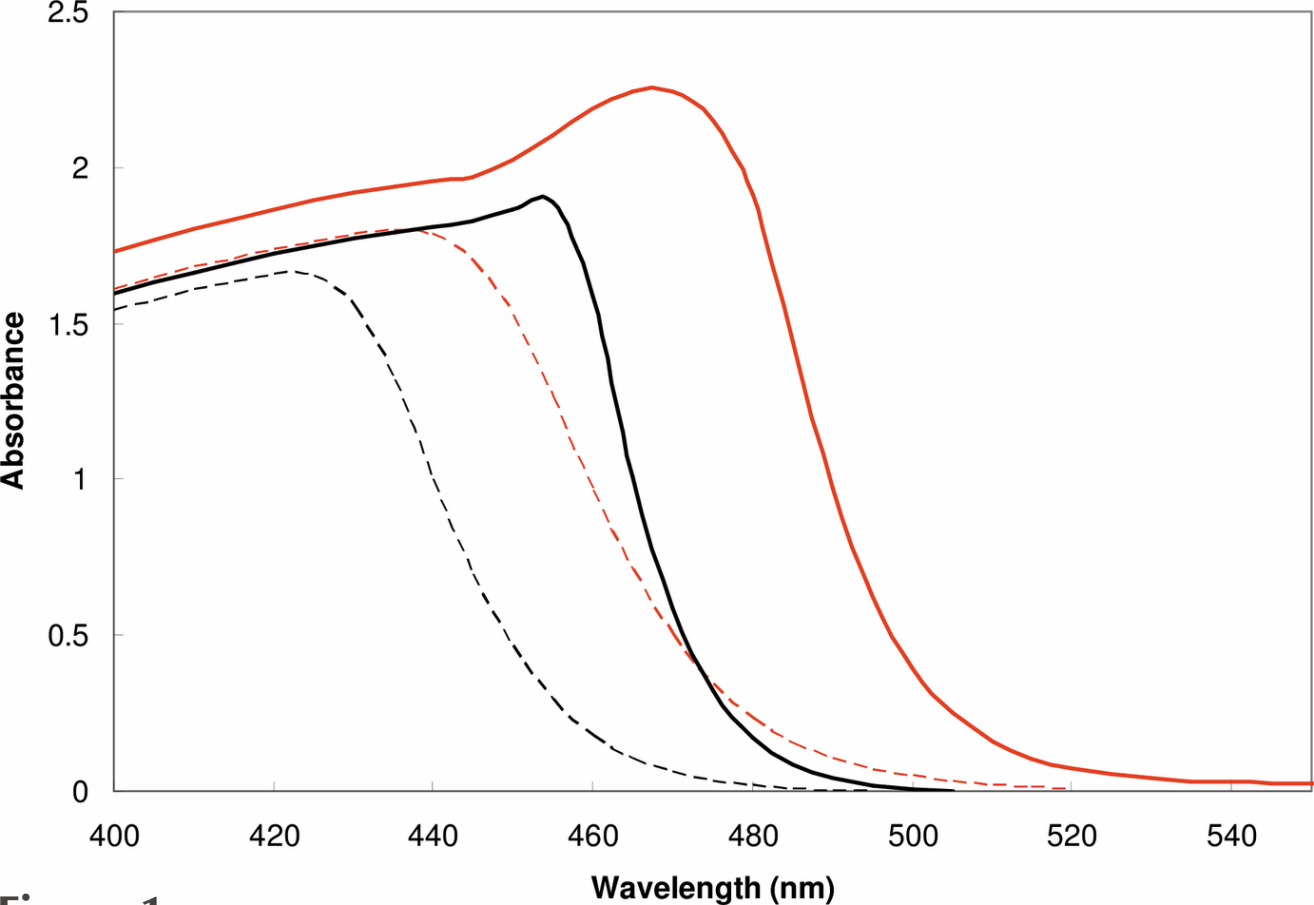
Visible region spectra for compound **1** (red), and compound **2** (black). Concentrations in acetone: solid, 20 m*M*, dashed, 2 m*M*.

In a prior communication from this laboratory (Filley, 2024 ▸), it was established that the crystal structure of 2-meth­oxy-5-nitro­aniline consists of hydrogen-bonded arrays of π-stacked mol­ecules with dipole moments oriented anti-parallel, where the hydrogen bonds are between both hydrogen atoms of the amine group and only one of the oxygen atoms of the nitro group. The close contacts of the aromatic rings, as well as the concentration-dependent red shift of the visible absorption spectra, were used to hypothesize that the orange–red color of the crystals, with light absorption near 490 nm, arises from an inter­molecular charge-transfer mechanism, similar to that seen in certain colored semiconductors. In order to probe the likelihood of this possibility, *N*-alkyl derivatives of 2-meth­oxy-5-nitro­aniline (hereinafter referred to as the parent) were prepared: 2-meth­oxy-*N*-(4-methyl­benz­yl)-5-nitro­aniline (**1**) and 2-meth­oxy-5-nitro-*N*-(tri­phenyl­methyl)­aniline (**2**). While the benzyl derivative forms orange crystals from ethanol, the tri­phenyl­methyl derivative forms yellow crystals, with both being suitable for single-crystal X-ray diffraction analysis. A UV-Vis analysis of both compounds reveals they have similar spectra in the UV region, with absorption maxima ranging from 380 to 392 nm (Table 1 ▸), but the orange benzyl derivative has concentration-dependent spectra in the visible region, fully analogous to the spectra reported for the parent compound. The results are displayed in Fig. 1 ▸. Aggregation of organic dyes, and the accompanying changes to spectral properties, is of inter­est to those seeking to advance the topic of organic electronics (González-Sánchez *et al.*, 2026 ▸; Heyne, 2016 ▸). Other work has focused on the effects of packing on fluorescence emission from crystals (Zhang *et al.*, 2022 ▸) and on the effects of co-crystallized mol­ecules (Tarai & Baruah, 2018 ▸). A motivation for making compounds **1** and **2** is the possibility that modifications of the parent could lead to compounds that will be suitable for specific needs or applications. In the realm of inorganic semiconductors, one is required to modify the crystal by modifying the elements or their composition, with no real anti­cipation of how the changes will affect the overall properties. If one contemplates exploiting the properties of the parent by modifying its mol­ecular structure, then data regarding how changes to the mol­ecule will affect its crystal structure and color become crucial. The results reported here show that modifying the amine with a benzyl group does not inter­fere with the ability of the aromatic rings of the aniline mol­ecules to approach each other and maintain the putative charge-transfer mechanism and color center formation. The more sterically demanding tri­phenyl­methyl group, on the other hand, does inter­fere with stacking and prevents formation of an orange color center. It is also noteworthy that the core color of both compounds arises from the tail of the UV spectrum into the visible region to give yellow solutions, and that aggregation and concentration-dependent spectral changes into regions of the visible spectrum close to 490 nm are only observed for the sterically unencumbered compound **1**. Using the suite of organic reactions available for modifying amines, it is possible to imagine a host of derivatives capable of binding to surfaces, forming polymers, taking on liquid crystalline properties, or partaking in amphiphilic inter­actions, to name a few, with due consideration of the steric demand around the amine nitro­gen atom. The results reported here could help inform future endeavors along these lines.
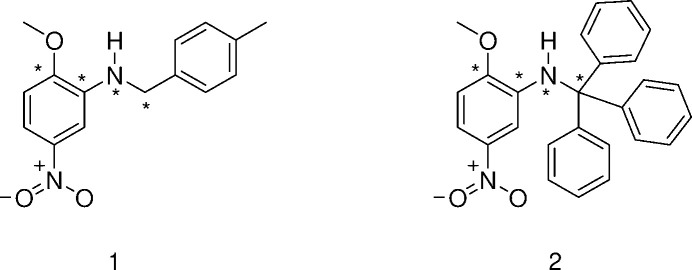


## 2. Structural commentary

**Figure 2:**
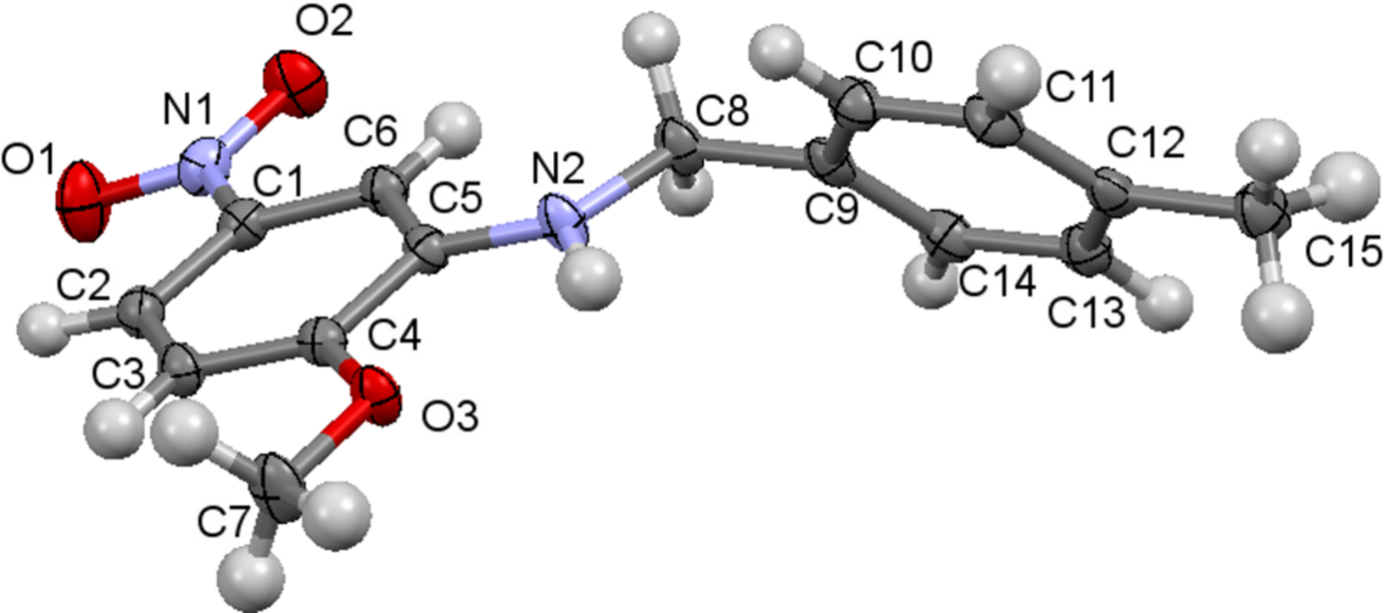
Mol­ecular structure of compound **1** with atoms displayed as displacement ellipsoids at the 50% probability level.

**Figure 3:**
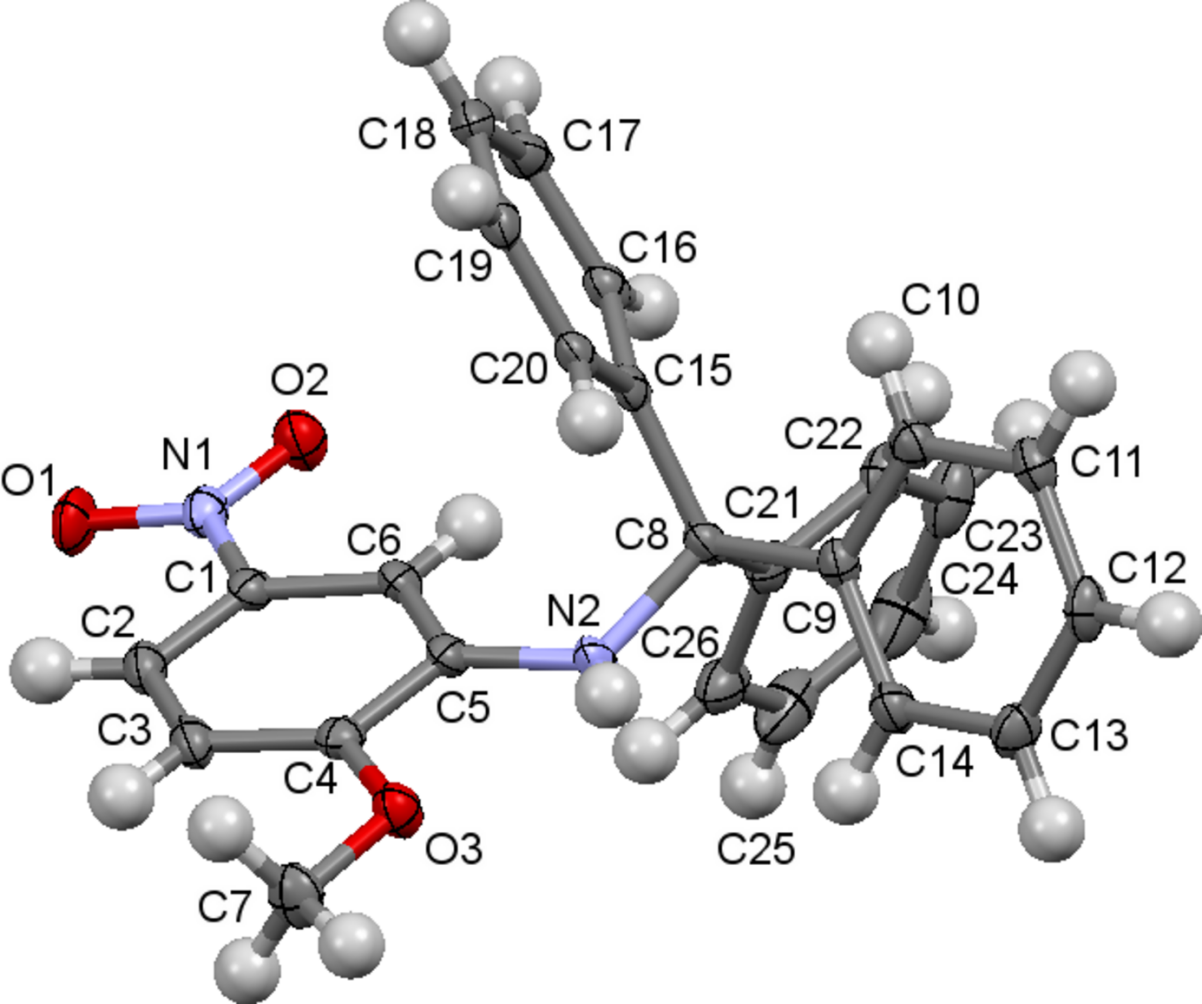
Molecular structure of the first crystallographically independent molecule of compound **2** with atoms displayed as displacement ellipsoids at the 50% probability level.

The molecular structure of compound **1** is shown in Fig. 2 ▸, and the two crystallographically independent molecules of compound **2** are shown in Figs. 3 ▸ and 4 ▸, respectively. Note that compound **2** crystallizes as two crystallographically independent mol­ecules, but the second mol­ecule is not displayed in Fig. 3 ▸ to help facilitate visual comparison to that of compound **1**. Compounds **1** and **2** adopt a conformation in which the attached alkyl group is *trans* with respect to the meth­oxy group about the aromatic-amine C—N bond, with similar crystallographically determined distances for both compounds of 2.23 (3) Å (O3⋯H2) and 4.04 (3) Å (O3⋯C8). This conformation is most likely favored due to steric effects. The near-planarity of the amine nitro­gen atom suggests some delocalization of the lone-pair electrons on the nitro­gen atom into the aromatic ring, although the amine group is *meta* to the nitro group and therefore not directly conjugated to it. The planarity of the nitro­gen atoms here is compared to that of the parent compound in Table 1 ▸. It is evident that the mol­ecule with its amine nitro­gen atom closest to planar (180°) is the less hindered compound **1**, while the tri­phenyl­methyl derivative and the parent have comparably pyramidal amine N atoms. Based on these data, it appears the main factor controlling the geometry at the amine nitro­gen atom is crystal packing for these compounds, and not strong electronic effects that contribute to, or prevent, delocalization of the nitro­gen lone pair.

## 3. Supra­molecular features

**Figure 5:**
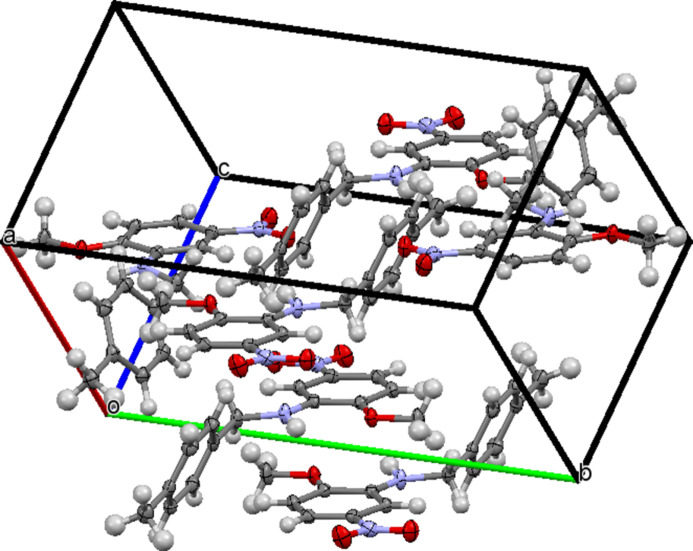
Unit cell and stacking arrangement in crystals of compound **1**.

The π-stacking observed for compound **1** is displayed in Fig. 5 ▸, which shows the unit cell and two additional mol­ecules at the bottom of the figure to highlight the stacking. While the central aromatic rings bearing the polar meth­oxy groups and the nitro groups stack in pairs with their dipole moments oriented anti-parallel, the next pair of mol­ecules is offset by an amount equivalent to the dimensions of the benzyl groups, which is about 6.5 Å. Pairs of face-to-face stacked benzyl groups stack against the central aromatic ring in an edge-to-face manner at an angle of 65.8 (3)°. A second set of benzyl groups exist, shown on the outermost mol­ecules to the right and left in Fig. 5 ▸, which also stack in a pair-wise fashion against the central aromatic rings at 66.2 (3)°, making the angle between the benzyl groups 48.6 (3)°. These angles constitute an isosceles triangle with the plane defined by the central aromatic ring as its base. All of the central aromatic rings are parallel in the crystal to within about 1°. This is in contrast to crystals of the parent compound, where each pair of π-stacked rings is face-to-face π-stacked to the next pair, and separate columns of stacked rings are angled with respect to each other. In addition, no hydrogen-bonding is observed here, while in the case of the parent compound the stacks are hydrogen-bonded together. For compound **1**, the hypothesized color center formation due to charge transfer could be facilitated by overlap of delocalized electrons on the benzyl groups with the π-system of the central rings.

**Figure 6:**
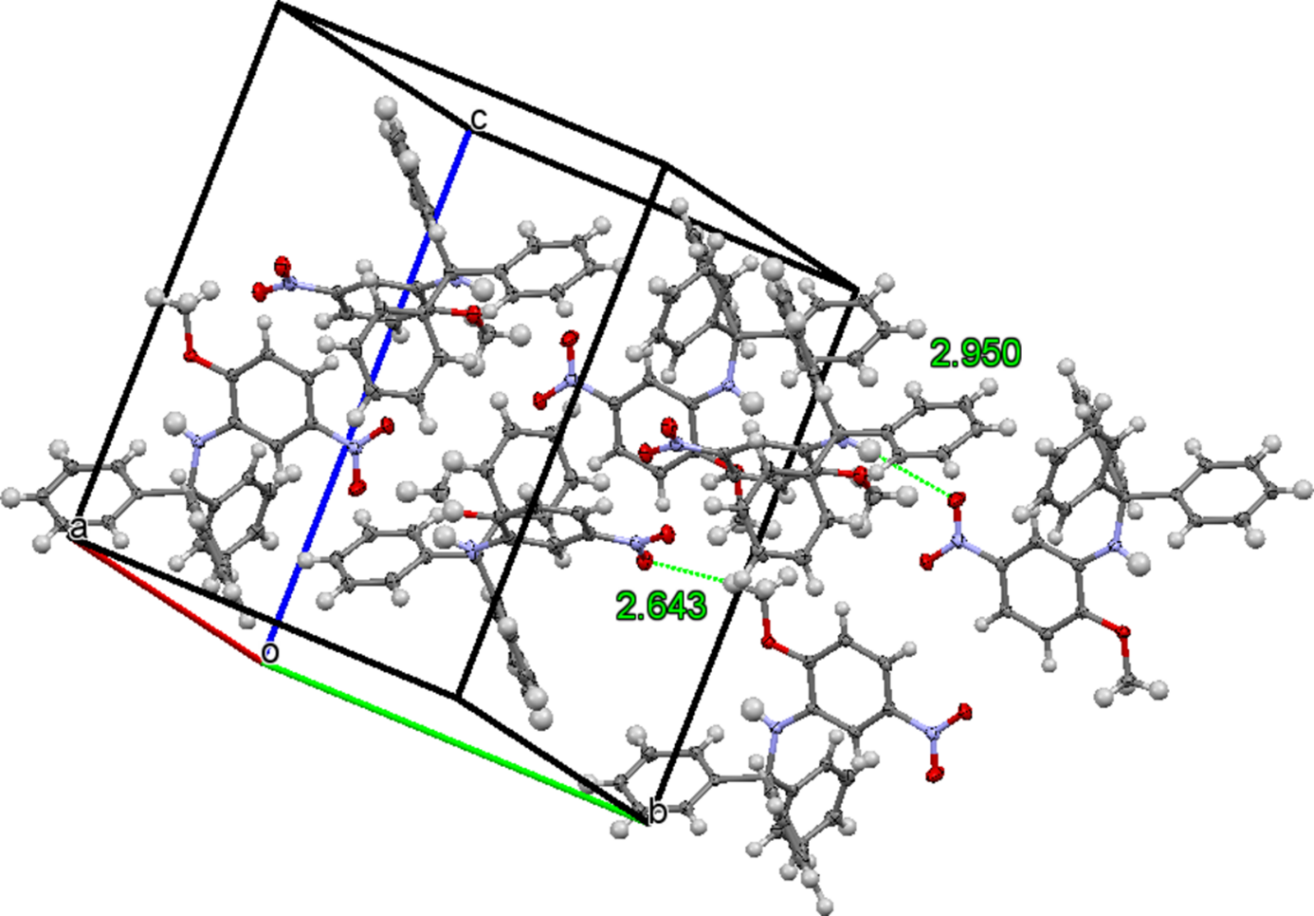
Unit cell and stacking arrangement in crystals of compound **2**.

The packing observed for compound **2** in Fig. 6 ▸, with the unit cell depicted as well as three additional mol­ecules, shows the lack of direct π-stacking between the central aromatic rings bearing the meth­oxy and the nitro groups, but does show the dipole moments are oriented anti-parallel. The two sets of crystallographically independent molecules are distinctive: one set has its nitro groups directed at each other but offset by about 3.1 Å, and the other set has molecules which lie side-by-side. The former set has nitro groups which are about 2.6 Å from a meth­oxy hydrogen atom, while the latter set has nitro groups which are about 2.9 Å from the aniline NH group, which constitutes a weak hydrogen bond for only one set of mol­ecules. The angle between the two sets of central aromatic rings is 49.4 (2)°. The sterically bulky tri­phenyl­methyl group prevents direct face-to-face contact of the central aromatic rings, which gives rise to the yellow color of the crystals. The lower wavelength absorption indicates there is a higher-energy charge-transfer mechanism in crystals of compound **2**, and further supports the existence of a more favorable charge-transfer process in crystals of compound **1**.

## 4. Database survey

The authors are unaware of examples in the literature of simple anilines which have been alkyl­ated with the express purpose of changing the crystal π-stacking arrangement, and as a result, the color of the crystal. A red anthracene derivative has been studied as a potential organic semiconductor with the emphasis on intra­molecular charge transfer (Zaini *et al.*, 2019 ▸). The stacking in these crystals could lend itself to inter­molecular charge transfer, but was not discussed by the authors. Crystals of *N*-(4-methyl­benz­yl)-3-nitro­aniline (Đaković *et al.*, 2012 ▸) show the mol­ecule has a near planar amino group with a C—C—N—C torsion angle of 179.2°. In spite of intra­molecular hydrogen bonding in an *N*-(4-methyl­benz­yl)-substituted amino­coumarin, the amino group participates in a C—C—N—C torsion angle of 171.8° (Rambabu *et al.*, 2010 ▸). Both these values can be compared to compound **1** in Table 1 ▸. In *N*-trityl-2-(tritylsulfan­yl)aniline, the amino group has a similar pyramidal N atom of the amino group to compound **2**, with no strong electron demand on the central aromatic ring (Neuba *et al.*, 2011 ▸). A tri­phenyl­methyl-substituted Schiff base crystallizes in two crystallographically independent mol­ecules (Theppitak *et al.*, 2014 ▸) similar to compound **2**, but with analogous torsion angles that differ much less from each other (138.6 and −137.1°) than those in Table 1 ▸.

## 5. Synthesis and crystallization

Reagents were obtained from Aldrich and used as received except 2-meth­oxy-5-nitro­aniline, which was recrystallized from 95% *v*/*v* ethanol. Tri­phenyl­methyl ­chloride was stored in a desiccator.

Compound **1**: A 9 ml vial was charged with 250 mg (1.5 mmol) 2-meth­oxy-5-nitro­aniline, 400 mg (4.8 mmol) NaHCO_3_, 2 ml tri­ethyl­eneglycol monomethyl ether and 220 µl (1.7 mmol) 4-methyl­benzyl chloride. The slurry was stirred and heated to 438±2 K for 5 min while monitoring with a thermocouple. The mixture was cooled to room temperature, poured into 30 ml water, and stirred at room temperature for 30 min. After filtration, the red filter cake was allowed to air dry; yield 275 mg (67%). This material was boiled in 10 ml 95% *v*/*v* ethanol, filtered, and allowed to crystallize overnight at 273 K. Filtration and washing with cold ethanol afforded orange crystals suitable for X-ray diffraction experiments; yield 125 mg (31%). ^1^H NMR (400 MHz, CDCl_3_): 2.40 (*s*, 3H), 3.98 (*s*, 3H), 4.39 (*s*, 2H), 4.80 (*bs*, 1H), 6.79–7.69 (*m*, 7H). ^13^C NMR (CDCl_3_): 21.1, 47.5, 56.0, 103.9, 107.9, 113.4, 127.9, 129.5, 135.1, 137.3, 138.2, 142.4, 151.6.

Compound **2**: A slurry of 250 mg (1.5 mmol) 2-meth­oxy-5-nitro­aniline and 500 mg (6.0 mmol) NaHCO_3_ in 5 ml acetone was treated at room temperature with solid tri­phenyl­methyl­chloride (415 mg, 1.5 mmol) with stirring. After 2 h the mixture turned lemon yellow. The mixture was poured into 30 ml water to produce a gooey solid which could be triturated with a little ethanol to give a granular solid. After stirring for 1 h, the solids were filtered off and air dried overnight to give a yellow solid; yield, 542 mg (89%). This material was dissolved by boiling in a mixture of 10 ml 95% *v*/*v* ethanol + 10 ml acetone and filtered. After boiling to reduce the volume by 10-20%, cooling and seeding gave yellow diamond-shaped crystals; yield 382 mg (62%). ^1^H NMR (400 MHz, CDCl_3_): 4.00 (*s*, 3H), 5.95 (*s*, 1H), 6.73–7.53 (*m*, 18H). ^13^C NMR (CDCl_3_): 56.2, 71.3, 107.6, 109.3, 113.3, 126.8, 127.8, 128.4, 136.0, 141.3, 144.5, 151.9.

## 6. Refinement

Details of data collection and structure refinement are given in Table 2 ▸. General refinement notes: Hydrogen atoms were inserted at idealized positions and refined using a riding model with isotropic parameters except for those on the amine nitro­gen atoms. The hydrogen atoms for the amine nitro­gen atoms were introduced with the AFIX 23 command in *SHELXL* (Sheldrick, 2015*b* ▸) and then one of the hydrogen atoms was deleted from each amine. The remaining hydrogen-atom positions were then refined freely while still being restrained to idealized bond lengths. Specific additional procedures for each compound are found below.

Compound **1**: To eliminate residual electron density on bonds, the refinement was completed using *NoSpherA2* using non-spherical scattering factors (Kleemiss *et al.*, 2021 ▸). The density functional theory calculations used to obtain mol­ecular wavefunctions were calculated using *ORCA 5.0* (Neese, 2012 ▸, 2022 ▸). The r2SCAN method was used with the cc-pVTZ basis set. Hydrogen atoms were refined isotropically.

Compound **2**: A solvent mask was employed to model the electron density within a void that was partially occupied by ethanol (the solvent in the crystallization). The void is on a special position and the solvent appears to be disordered: 29 electrons were found in a volume of 116 Å^3^ in one void per unit cell. This is consistent with the presence of 0.5(C_2_H_6_O) per asymmetric unit, which accounts for 52 electrons per unit cell. Hence, there are 0.25 ethanol mol­ecules per one mol­ecule of analyte.

## Supplementary Material

Crystal structure: contains datablock(s) 2, 1. DOI: 10.1107/S205698902600681X/wm5804sup1.cif

Structure factors: contains datablock(s) 2. DOI: 10.1107/S205698902600681X/wm58042sup3.hkl

Structure factors: contains datablock(s) 1. DOI: 10.1107/S205698902600681X/wm58041sup2.hkl

Supporting information file. DOI: 10.1107/S205698902600681X/wm58042sup4.cml

Supporting information file. DOI: 10.1107/S205698902600681X/wm58041sup5.cml

CCDC references: 2546245, 2546246

Additional supporting information:  crystallographic information; 3D view; checkCIF report

## Figures and Tables

**Figure 4:**
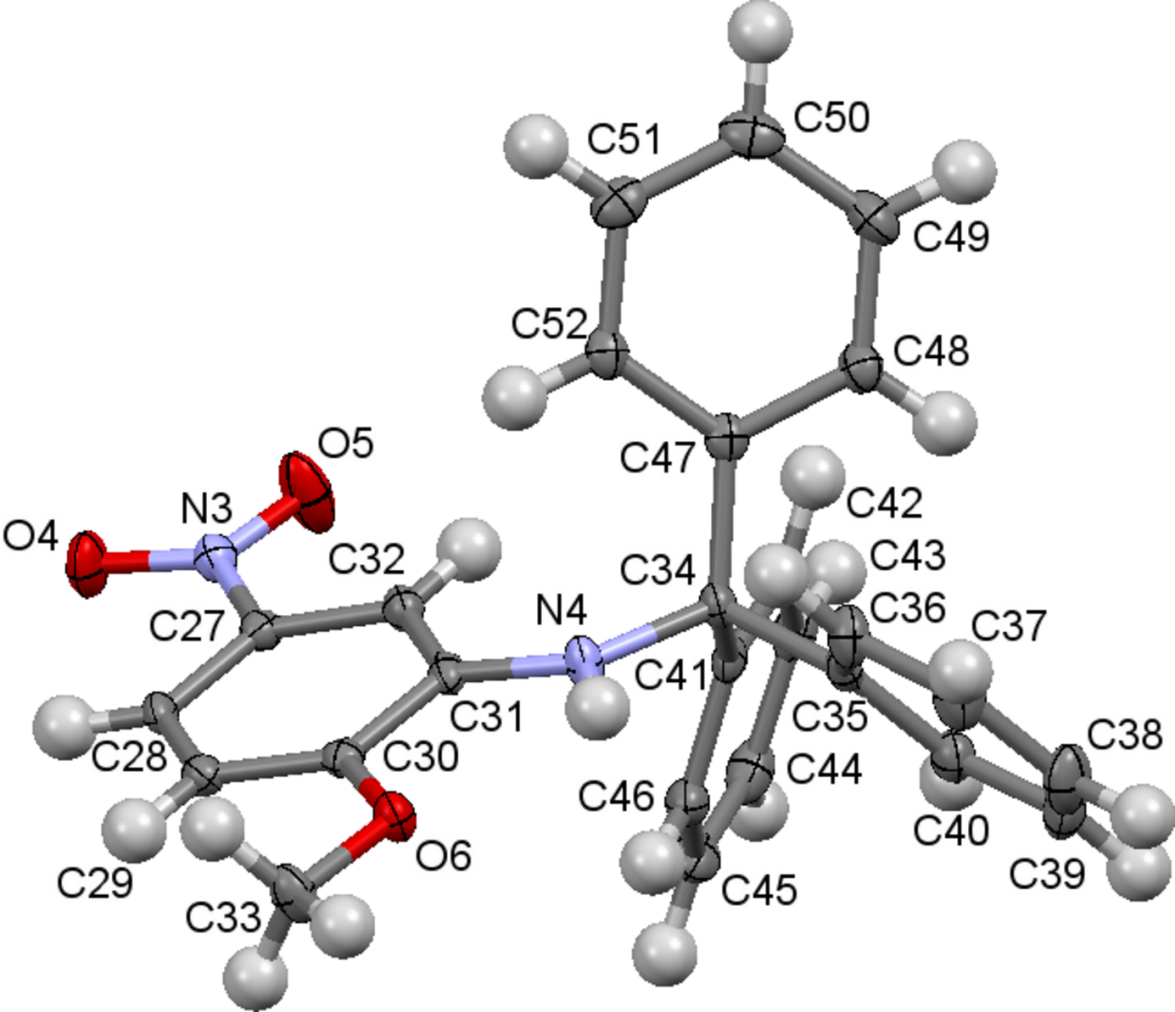
Molecular structure of the second crystallographically independent molecule of compound **2** with atoms displayed as displacement ellipsoids at the 50% probability level.

**Table 1:** Planarity of amine nitro­gen atoms for the compounds considered here Torsion angles using the four atoms indicated in the Scheme. Uncertainties are +/- 0.2°. Spectroscopic data for each compound dissolved in acetone, 0.1 m*M*.

Compound	Atoms used	Torsion angle	λ_max_ (nm)
**1**	C4—C5—N2—C8	172.31 (13)°	392
**2**	C4—C5—N2—C8, C30—C31—N4—C34^*a*,*b*^	–157.32 (19)°, 168.5 (2)°	383
Parent^*c*^	C2—C1—N1—H1B	–159.3°	380

Note: (*a*). The second value is for the second crystallographically independent mol­ecule. (*b*). The authors are indebted to a reviewer for pointing out the only other significant differences between the independent mol­ecules are in the tri­phenyl­methyl groups. (*c*). Taken from Filley (2024 ▸).

**Table 2:** Experimental details

	**1**	**2**
Crystal data		
Chemical formula	C_15_H_16_N_2_O_3_	2C_26_H_22_N_2_O_3_·0.5C_2_H_6_O
*M*_r_	272.31	843.99
Crystal system, space group	Monoclinic, *P*2_1_/*c*	Triclinic, *P* 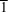
Temperature (K)	130	100
*a*, *b*, *c* (Å)	9.5017 (4), 15.7855 (7), 8.9908 (4)	9.0068 (3), 14.1142 (5), 18.2135 (6)
α, β, γ (°)	90, 95.230 (2), 90	103.152 (2), 98.267 (2), 106.000 (2)
*V* (Å^3^)	1342.91 (10)	2113.90 (13)
*Z*	4	2
Radiation type	Mo *K*α	Mo *K*α
μ (mm^−1^)	0.10	0.09
Crystal size (mm)	0.29 × 0.17 × 0.05	0.14 × 0.05 × 0.03

Data collection		
Diffractometer	Bruker APEXII CCD	Bruker APEXII CCD
Absorption correction	Multi-scan (*SADABS*; Krause *et al.*, 2015 ▸)	Multi-scan (*SADABS*; Krause *et al.*, 2015 ▸)
*T*_min_, *T*_max_	0.657, 0.746	0.654, 0.745
No. of measured, independent and observed [*I* ≥ 2u(*I*)] reflections	30573, 3078, 2497	35447, 8768, 6044
*R*_int_	0.050	0.065
(sin θ/λ)_max_ (Å^−1^)	0.649	0.628

Refinement		
*R*[*F*^2^ > 2σ(*F*^2^)], *wR*(*F*^2^), *S*	0.048, 0.136, 1.08	0.051, 0.120, 1.04
No. of reflections	3078	8768
No. of parameters	245	568
H-atom treatment	All H-atom parameters refined	H atoms treated by a mixture of independent and constrained refinement
Δρ_max_, Δρ_min_ (e Å^−3^)	0.33, −0.38	0.40, −0.41

Computer programs: *APEX5* and *SAINT* (Bruker, 2024 ▸), *OLEX2.solve* and *OLEX2.refine* (Bourhis *et al.*, 2015 ▸), *SHELXT* (Sheldrick, 2015*a* ▸), *OLEX2* (Dolomanov *et al.*, 2009 ▸) and *publCIF* (Westrip, 2010 ▸).

## supplementary crystallographic information

### 2-Methoxy-5-nitro-N-(triphenylmethyl)aniline ethanol quatersolvate (2) Crystal data

**Table:**

2C_26_H_22_N_2_O_3_·0.5C_2_H_6_O	*Z* = 2
*M**_r_* = 843.99	*F*(000) = 890.568
Triclinic, *P*1	*D*_x_ = 1.326 Mg m^−^^3^
*a* = 9.0068 (3) Å	Mo *K*α radiation, λ = 0.71073 Å
*b* = 14.1142 (5) Å	Cell parameters from 8823 reflections
*c* = 18.2135 (6) Å	θ = 2.4–26.4°
α = 103.152 (2)°	µ = 0.09 mm^−^^1^
β = 98.267 (2)°	*T* = 100 K
γ = 106.000 (2)°	Plate, clear light yellow
*V* = 2113.90 (13) Å^3^	0.14 × 0.05 × 0.03 mm

### 2-Methoxy-5-nitro-N-(triphenylmethyl)aniline ethanol quatersolvate (2) Data collection

**Table:**

Bruker APEXII CCD diffractometer	6044 reflections with *I*≥ 2u(*I*)
φ and ω scans	*R*_int_ = 0.065
Absorption correction: multi-scan (*SADABS*; Krause *et al.*, 2015)	θ_max_ = 26.5°, θ_min_ = 1.6°
*T*_min_ = 0.654, *T*_max_ = 0.745	*h* = −11→11
35447 measured reflections	*k* = −17→17
8768 independent reflections	*l* = −22→22

### 2-Methoxy-5-nitro-N-(triphenylmethyl)aniline ethanol quatersolvate (2) Refinement

**Table:**

Refinement on *F*^2^	82 constraints
Least-squares matrix: full	H atoms treated by a mixture of independent and constrained refinement
*R*[*F*^2^ > 2σ(*F*^2^)] = 0.051	*w* = 1/[σ^2^(*F*_o_^2^) + (0.0403*P*)^2^ + 1.2162*P*] where *P* = (*F*_o_^2^ + 2*F*_c_^2^)/3
*wR*(*F*^2^) = 0.120	(Δ/σ)_max_ = 0.0004
*S* = 1.04	Δρ_max_ = 0.40 e Å^−^^3^
8768 reflections	Δρ_min_ = −0.41 e Å^−^^3^
568 parameters	Extinction correction: olex2.refine (Bourhis *et al.*, 2015), Fc^*^=kFc[1+0.001xFc^2^λ^3^/sin(2θ)]^-1/4^
0 restraints	Extinction coefficient: 0.0061 (7)

### 2-Methoxy-5-nitro-N-(triphenylmethyl)aniline ethanol quatersolvate (2) Fractional atomic coordinates and isotropic or equivalent isotropic displacement parameters (Å^2^)

**Table:**

	*x*	*y*	*z*	*U*_iso_*/*U*_eq_	
O6	−0.05185 (16)	0.86210 (10)	0.63110 (8)	0.0188 (3)	
O5	0.2701 (2)	0.54837 (12)	0.70856 (10)	0.0403 (5)	
O4	0.11966 (19)	0.45576 (11)	0.59889 (8)	0.0288 (4)	
N4	0.1770 (2)	0.89214 (13)	0.74752 (9)	0.0189 (4)	
N3	0.1689 (2)	0.53550 (13)	0.65170 (10)	0.0203 (4)	
C34	0.2845 (2)	0.92603 (14)	0.82444 (11)	0.0165 (4)	
C35	0.2581 (2)	1.02686 (15)	0.86641 (11)	0.0171 (4)	
C36	0.2895 (3)	1.10858 (15)	0.83358 (12)	0.0236 (5)	
H36	0.3262 (3)	1.10157 (15)	0.78693 (12)	0.0283 (6)*	
C37	0.2669 (3)	1.20014 (17)	0.86905 (13)	0.0312 (6)	
H37	0.2857 (3)	1.25488 (17)	0.84578 (13)	0.0375 (7)*	
C38	0.2172 (3)	1.21211 (17)	0.93803 (13)	0.0312 (5)	
H38	0.2028 (3)	1.27504 (17)	0.96232 (13)	0.0375 (7)*	
C39	0.1886 (3)	1.13228 (17)	0.97133 (12)	0.0273 (5)	
H39	0.1551 (3)	1.14043 (17)	1.01887 (12)	0.0328 (6)*	
C40	0.2085 (2)	1.03972 (16)	0.93556 (11)	0.0216 (5)	
H40	0.1879 (2)	0.98494 (16)	0.95874 (11)	0.0260 (5)*	
C47	0.4591 (2)	0.95285 (14)	0.81814 (11)	0.0167 (4)	
C48	0.5783 (2)	1.01270 (15)	0.88356 (11)	0.0200 (4)	
H48	0.5504 (2)	1.03492 (15)	0.93140 (11)	0.0241 (5)*	
C49	0.7362 (3)	1.03999 (16)	0.87956 (12)	0.0241 (5)	
H49	0.8155 (3)	1.08026 (16)	0.92463 (12)	0.0290 (6)*	
C50	0.7793 (3)	1.00897 (16)	0.81028 (13)	0.0266 (5)	
H50	0.8877 (3)	1.02721 (16)	0.80765 (13)	0.0319 (6)*	
C51	0.6627 (3)	0.95120 (16)	0.74510 (12)	0.0242 (5)	
H51	0.6913 (3)	0.93016 (16)	0.69726 (12)	0.0290 (6)*	
C52	0.5041 (2)	0.92349 (15)	0.74876 (11)	0.0194 (4)	
H52	0.4253 (2)	0.88402 (15)	0.70329 (11)	0.0233 (5)*	
C41	0.2371 (2)	0.84455 (14)	0.86806 (11)	0.0173 (4)	
C42	0.3467 (3)	0.82005 (15)	0.91590 (11)	0.0197 (4)	
H42	0.4567 (3)	0.85281 (15)	0.92159 (11)	0.0236 (5)*	
C43	0.2966 (3)	0.74791 (16)	0.95556 (12)	0.0242 (5)	
H43	0.3727 (3)	0.73284 (16)	0.98890 (12)	0.0291 (6)*	
C44	0.1374 (3)	0.69810 (16)	0.94685 (12)	0.0270 (5)	
H44	0.1039 (3)	0.64784 (16)	0.97318 (12)	0.0324 (6)*	
C45	0.0268 (3)	0.72190 (16)	0.89944 (12)	0.0247 (5)	
H45	−0.0831 (3)	0.68799 (16)	0.89318 (12)	0.0296 (6)*	
C46	0.0765 (2)	0.79508 (15)	0.86126 (11)	0.0212 (5)	
H46	−0.0002 (2)	0.81202 (15)	0.82978 (11)	0.0254 (5)*	
C31	0.1201 (2)	0.79496 (15)	0.69631 (11)	0.0168 (4)	
C30	−0.0050 (2)	0.77825 (14)	0.63240 (11)	0.0157 (4)	
C29	−0.0699 (2)	0.68361 (15)	0.57784 (11)	0.0176 (4)	
H29	−0.1521 (2)	0.67426 (15)	0.53519 (11)	0.0211 (5)*	
C28	−0.0164 (2)	0.60232 (15)	0.58474 (11)	0.0178 (4)	
H28	−0.0627 (2)	0.53666 (15)	0.54819 (11)	0.0213 (5)*	
C27	0.1062 (2)	0.61928 (14)	0.64617 (11)	0.0163 (4)	
C32	0.1759 (2)	0.71389 (15)	0.70176 (11)	0.0183 (4)	
H32	0.2608 (2)	0.72280 (15)	0.74298 (11)	0.0219 (5)*	
C33	−0.1686 (3)	0.85354 (16)	0.56545 (12)	0.0240 (5)	
H33a	−0.1305 (8)	0.8348 (11)	0.51819 (14)	0.0360 (7)*	
H33b	−0.2674 (6)	0.8005 (8)	0.5630 (5)	0.0360 (7)*	
H33c	−0.1878 (13)	0.9195 (3)	0.5703 (4)	0.0360 (7)*	
O3	0.16017 (17)	0.25537 (11)	0.69151 (8)	0.0245 (3)	
O2	0.67154 (18)	0.06046 (11)	0.60283 (9)	0.0287 (4)	
O1	0.46758 (19)	−0.07656 (11)	0.56056 (9)	0.0297 (4)	
N2	0.4656 (2)	0.34466 (13)	0.71727 (10)	0.0230 (4)	
N1	0.5281 (2)	0.01637 (13)	0.59118 (10)	0.0225 (4)	
C8	0.6181 (2)	0.41256 (15)	0.71099 (12)	0.0197 (4)	
C9	0.6202 (2)	0.52349 (15)	0.74515 (12)	0.0192 (4)	
C10	0.6793 (2)	0.60141 (15)	0.71176 (12)	0.0207 (4)	
H10	0.7179 (2)	0.58612 (15)	0.66608 (12)	0.0248 (5)*	
C11	0.6826 (2)	0.70096 (15)	0.74425 (12)	0.0228 (5)	
H11	0.7214 (2)	0.75298 (15)	0.72019 (12)	0.0274 (6)*	
C12	0.6298 (3)	0.72491 (16)	0.81120 (12)	0.0244 (5)	
H12	0.6316 (3)	0.79315 (16)	0.83320 (12)	0.0293 (6)*	
C13	0.5742 (3)	0.64900 (16)	0.84627 (13)	0.0261 (5)	
H13	0.5389 (3)	0.66523 (16)	0.89284 (13)	0.0313 (6)*	
C14	0.5700 (3)	0.54874 (15)	0.81331 (12)	0.0234 (5)	
H14	0.5323 (3)	0.49707 (15)	0.83784 (12)	0.0280 (6)*	
C21	0.7579 (3)	0.40003 (15)	0.76359 (12)	0.0227 (5)	
C22	0.9109 (3)	0.46387 (16)	0.77062 (12)	0.0253 (5)	
H22	0.9277 (3)	0.51224 (16)	0.74144 (12)	0.0303 (6)*	
C23	1.0391 (3)	0.45803 (18)	0.81951 (14)	0.0362 (6)	
H23	1.1428 (3)	0.50184 (18)	0.82349 (14)	0.0435 (7)*	
C24	1.0152 (4)	0.3881 (2)	0.86250 (15)	0.0441 (7)	
H24	1.1027 (4)	0.3838 (2)	0.89605 (15)	0.0530 (8)*	
H4	0.132 (3)	0.937 (2)	0.7371 (16)	0.0530 (8)*	
H2	0.391 (3)	0.377 (2)	0.7204 (16)	0.0530 (8)*	
C25	0.8645 (4)	0.32474 (19)	0.85655 (14)	0.0424 (7)	
H25	0.8484 (4)	0.27654 (19)	0.88588 (14)	0.0508 (8)*	
C26	0.7358 (3)	0.33096 (17)	0.80789 (13)	0.0309 (5)	
H26	0.6321 (3)	0.28779 (17)	0.80484 (13)	0.0371 (7)*	
C15	0.6266 (2)	0.39124 (14)	0.62520 (12)	0.0188 (4)	
C16	0.7381 (2)	0.35243 (15)	0.59517 (12)	0.0208 (4)	
H16	0.8210 (2)	0.34427 (15)	0.62920 (12)	0.0250 (5)*	
C17	0.7298 (3)	0.32537 (16)	0.51600 (13)	0.0258 (5)	
H17	0.8052 (3)	0.29715 (16)	0.49632 (13)	0.0310 (6)*	
C18	0.6129 (3)	0.33928 (16)	0.46598 (12)	0.0263 (5)	
H18	0.6073 (3)	0.32081 (16)	0.41190 (12)	0.0315 (6)*	
C19	0.5037 (3)	0.38041 (15)	0.49526 (12)	0.0246 (5)	
H19	0.4248 (3)	0.39219 (15)	0.46121 (12)	0.0295 (6)*	
C20	0.5085 (2)	0.40457 (15)	0.57389 (12)	0.0208 (4)	
H20	0.4306 (2)	0.43049 (15)	0.59303 (12)	0.0249 (5)*	
C5	0.4060 (2)	0.24086 (15)	0.67691 (11)	0.0197 (4)	
C4	0.2406 (3)	0.19186 (15)	0.66358 (11)	0.0207 (4)	
C3	0.1729 (3)	0.08827 (16)	0.62569 (12)	0.0238 (5)	
H3	0.0615 (3)	0.05735 (16)	0.61644 (12)	0.0285 (6)*	
C2	0.2658 (3)	0.02902 (16)	0.60106 (12)	0.0230 (5)	
H2a	0.2198 (3)	−0.04222 (16)	0.57532 (12)	0.0276 (6)*	
C1	0.4264 (2)	0.07654 (15)	0.61505 (11)	0.0195 (4)	
C6	0.4986 (3)	0.18088 (15)	0.65251 (12)	0.0215 (5)	
H6	0.6101 (3)	0.21076 (15)	0.66129 (12)	0.0258 (5)*	
C7	−0.0070 (3)	0.21186 (18)	0.68010 (14)	0.0299 (5)	
H7a	−0.0547 (3)	0.1865 (11)	0.62459 (14)	0.0448 (8)*	
H7b	−0.0308 (3)	0.1548 (8)	0.7033 (8)	0.0448 (8)*	
H7c	−0.0505 (3)	0.2644 (4)	0.7045 (8)	0.0448 (8)*	

### 2-Methoxy-5-nitro-N-(triphenylmethyl)aniline ethanol quatersolvate (2) Atomic displacement parameters (Å^2^)

**Table:**

	*U*^11^	*U*^22^	*U*^33^	*U*^12^	*U*^13^	*U*^23^
O6	0.0195 (7)	0.0163 (7)	0.0175 (7)	0.0048 (6)	−0.0009 (6)	0.0028 (5)
O5	0.0435 (11)	0.0264 (9)	0.0399 (10)	0.0161 (8)	−0.0183 (8)	−0.0008 (7)
O4	0.0369 (9)	0.0187 (8)	0.0250 (8)	0.0111 (7)	−0.0002 (7)	−0.0030 (6)
N4	0.0222 (9)	0.0148 (8)	0.0158 (8)	0.0056 (7)	−0.0025 (7)	0.0013 (7)
N3	0.0192 (9)	0.0180 (9)	0.0201 (9)	0.0037 (7)	0.0021 (7)	0.0024 (7)
C34	0.0174 (10)	0.0148 (10)	0.0142 (9)	0.0043 (8)	−0.0001 (8)	0.0015 (7)
C35	0.0146 (10)	0.0159 (10)	0.0166 (10)	0.0048 (8)	−0.0030 (8)	0.0003 (8)
C36	0.0323 (12)	0.0197 (11)	0.0160 (10)	0.0074 (9)	0.0013 (9)	0.0032 (8)
C37	0.0451 (15)	0.0219 (12)	0.0262 (12)	0.0133 (11)	0.0001 (11)	0.0074 (9)
C38	0.0430 (15)	0.0229 (12)	0.0270 (12)	0.0181 (11)	0.0015 (10)	0.0000 (9)
C39	0.0319 (13)	0.0275 (12)	0.0192 (11)	0.0112 (10)	0.0044 (9)	−0.0010 (9)
C40	0.0232 (11)	0.0215 (11)	0.0180 (10)	0.0074 (9)	0.0015 (8)	0.0029 (8)
C47	0.0205 (11)	0.0124 (9)	0.0176 (10)	0.0049 (8)	0.0038 (8)	0.0060 (8)
C48	0.0218 (11)	0.0201 (10)	0.0178 (10)	0.0058 (9)	0.0033 (8)	0.0063 (8)
C49	0.0202 (11)	0.0212 (11)	0.0259 (11)	0.0008 (9)	−0.0013 (9)	0.0078 (9)
C50	0.0222 (12)	0.0253 (12)	0.0360 (13)	0.0062 (9)	0.0082 (10)	0.0166 (10)
C51	0.0293 (12)	0.0228 (11)	0.0262 (11)	0.0101 (9)	0.0125 (9)	0.0117 (9)
C52	0.0237 (11)	0.0145 (10)	0.0191 (10)	0.0048 (9)	0.0035 (8)	0.0050 (8)
C41	0.0218 (11)	0.0131 (10)	0.0137 (9)	0.0043 (8)	0.0037 (8)	−0.0007 (7)
C42	0.0225 (11)	0.0178 (10)	0.0193 (10)	0.0078 (9)	0.0067 (8)	0.0032 (8)
C43	0.0352 (13)	0.0221 (11)	0.0177 (10)	0.0121 (10)	0.0069 (9)	0.0059 (8)
C44	0.0416 (14)	0.0170 (11)	0.0230 (11)	0.0063 (10)	0.0142 (10)	0.0060 (9)
C45	0.0240 (12)	0.0195 (11)	0.0254 (11)	−0.0002 (9)	0.0086 (9)	0.0030 (9)
C46	0.0205 (11)	0.0209 (11)	0.0187 (10)	0.0044 (9)	0.0042 (8)	0.0017 (8)
C31	0.0167 (10)	0.0161 (10)	0.0131 (9)	−0.0004 (8)	0.0037 (8)	0.0019 (8)
C30	0.0146 (10)	0.0163 (10)	0.0166 (10)	0.0038 (8)	0.0056 (8)	0.0055 (8)
C29	0.0152 (10)	0.0192 (10)	0.0165 (10)	0.0038 (8)	0.0027 (8)	0.0039 (8)
C28	0.0159 (10)	0.0151 (10)	0.0163 (10)	0.0004 (8)	0.0029 (8)	−0.0013 (8)
C27	0.0165 (10)	0.0136 (9)	0.0185 (10)	0.0038 (8)	0.0057 (8)	0.0044 (8)
C32	0.0182 (10)	0.0180 (10)	0.0158 (10)	0.0037 (8)	0.0019 (8)	0.0030 (8)
C33	0.0244 (12)	0.0201 (11)	0.0232 (11)	0.0065 (9)	−0.0045 (9)	0.0049 (9)
O3	0.0236 (8)	0.0186 (7)	0.0306 (8)	0.0057 (6)	0.0082 (6)	0.0055 (6)
O2	0.0256 (9)	0.0227 (8)	0.0357 (9)	0.0057 (7)	0.0078 (7)	0.0057 (7)
O1	0.0381 (10)	0.0139 (8)	0.0311 (9)	0.0066 (7)	0.0040 (7)	−0.0009 (6)
N2	0.0233 (10)	0.0130 (9)	0.0303 (10)	0.0014 (7)	0.0132 (8)	0.0024 (7)
N1	0.0302 (11)	0.0165 (9)	0.0191 (9)	0.0057 (8)	0.0039 (8)	0.0049 (7)
C8	0.0188 (11)	0.0142 (10)	0.0257 (11)	0.0030 (8)	0.0091 (9)	0.0053 (8)
C9	0.0159 (10)	0.0140 (10)	0.0248 (11)	0.0031 (8)	0.0033 (8)	0.0027 (8)
C10	0.0178 (11)	0.0182 (10)	0.0237 (11)	0.0037 (8)	0.0055 (8)	0.0032 (8)
C11	0.0213 (11)	0.0154 (10)	0.0292 (12)	0.0030 (9)	0.0034 (9)	0.0062 (9)
C12	0.0240 (12)	0.0145 (10)	0.0303 (12)	0.0069 (9)	0.0016 (9)	−0.0003 (9)
C13	0.0266 (12)	0.0222 (11)	0.0265 (12)	0.0084 (9)	0.0077 (9)	−0.0006 (9)
C14	0.0237 (11)	0.0170 (10)	0.0275 (11)	0.0033 (9)	0.0080 (9)	0.0049 (9)
C21	0.0336 (13)	0.0140 (10)	0.0204 (11)	0.0083 (9)	0.0074 (9)	0.0026 (8)
C22	0.0296 (12)	0.0167 (11)	0.0269 (12)	0.0086 (9)	0.0020 (9)	0.0025 (9)
C23	0.0375 (15)	0.0266 (13)	0.0365 (14)	0.0139 (11)	−0.0050 (11)	−0.0031 (10)
C24	0.0617 (19)	0.0322 (14)	0.0345 (14)	0.0269 (14)	−0.0113 (13)	0.0007 (11)
C25	0.078 (2)	0.0257 (13)	0.0254 (13)	0.0236 (14)	0.0033 (13)	0.0074 (10)
C26	0.0480 (15)	0.0200 (11)	0.0238 (11)	0.0098 (11)	0.0094 (11)	0.0049 (9)
C15	0.0210 (11)	0.0100 (9)	0.0237 (10)	0.0019 (8)	0.0075 (8)	0.0041 (8)
C16	0.0209 (11)	0.0141 (10)	0.0278 (11)	0.0041 (8)	0.0081 (9)	0.0066 (8)
C17	0.0281 (12)	0.0191 (11)	0.0316 (12)	0.0061 (9)	0.0167 (10)	0.0052 (9)
C18	0.0310 (13)	0.0190 (11)	0.0215 (11)	−0.0020 (9)	0.0090 (9)	0.0020 (9)
C19	0.0225 (12)	0.0175 (10)	0.0271 (12)	−0.0011 (9)	0.0003 (9)	0.0060 (9)
C20	0.0175 (11)	0.0142 (10)	0.0288 (11)	0.0012 (8)	0.0085 (9)	0.0055 (8)
C5	0.0255 (11)	0.0149 (10)	0.0186 (10)	0.0035 (8)	0.0084 (9)	0.0057 (8)
C4	0.0265 (12)	0.0198 (10)	0.0174 (10)	0.0069 (9)	0.0076 (9)	0.0072 (8)
C3	0.0244 (12)	0.0187 (11)	0.0237 (11)	0.0009 (9)	0.0020 (9)	0.0066 (9)
C2	0.0277 (12)	0.0156 (10)	0.0211 (11)	0.0019 (9)	0.0022 (9)	0.0043 (8)
C1	0.0260 (11)	0.0157 (10)	0.0181 (10)	0.0060 (9)	0.0080 (9)	0.0063 (8)
C6	0.0265 (12)	0.0151 (10)	0.0215 (10)	0.0012 (9)	0.0097 (9)	0.0063 (8)
C7	0.0196 (12)	0.0292 (12)	0.0385 (13)	0.0069 (10)	0.0057 (10)	0.0065 (10)

### 2-Methoxy-5-nitro-N-(triphenylmethyl)aniline ethanol quatersolvate (2) Geometric parameters (Å, º)

**Table:**

O6—C30	1.365 (2)	O3—C4	1.357 (2)
O6—C33	1.433 (2)	O3—C7	1.424 (3)
O5—N3	1.220 (2)	O2—N1	1.233 (2)
O4—N3	1.224 (2)	O1—N1	1.230 (2)
N4—C34	1.476 (2)	N2—C8	1.479 (3)
N4—C31	1.381 (2)	N2—H2	0.91 (3)
N4—H4	0.88 (3)	N2—C5	1.392 (2)
N3—C27	1.460 (3)	N1—C1	1.457 (3)
C34—C35	1.552 (3)	C8—C9	1.541 (3)
C34—C47	1.541 (3)	C8—C21	1.545 (3)
C34—C41	1.542 (3)	C8—C15	1.540 (3)
C35—C36	1.397 (3)	C9—C10	1.392 (3)
C35—C40	1.386 (3)	C9—C14	1.386 (3)
C36—H36	0.9500	C10—H10	0.9500
C36—C37	1.390 (3)	C10—C11	1.385 (3)
C37—H37	0.9500	C11—H11	0.9500
C37—C38	1.384 (3)	C11—C12	1.376 (3)
C38—H38	0.9500	C12—H12	0.9500
C38—C39	1.378 (3)	C12—C13	1.384 (3)
C39—H39	0.9500	C13—H13	0.9500
C39—C40	1.392 (3)	C13—C14	1.393 (3)
C40—H40	0.9500	C14—H14	0.9500
C47—C48	1.400 (3)	C21—C22	1.392 (3)
C47—C52	1.391 (3)	C21—C26	1.392 (3)
C48—H48	0.9500	C22—H22	0.9500
C48—C49	1.385 (3)	C22—C23	1.387 (3)
C49—H49	0.9500	C23—H23	0.9500
C49—C50	1.385 (3)	C23—C24	1.384 (4)
C50—H50	0.9500	C24—H24	0.9500
C50—C51	1.380 (3)	C24—C25	1.378 (4)
C51—H51	0.9500	C25—H25	0.9500
C51—C52	1.390 (3)	C25—C26	1.390 (4)
C52—H52	0.9500	C26—H26	0.9500
C41—C42	1.390 (3)	C15—C16	1.389 (3)
C41—C46	1.396 (3)	C15—C20	1.395 (3)
C42—H42	0.9500	C16—H16	0.9500
C42—C43	1.392 (3)	C16—C17	1.390 (3)
C43—H43	0.9500	C17—H17	0.9500
C43—C44	1.380 (3)	C17—C18	1.377 (3)
C44—H44	0.9500	C18—H18	0.9500
C44—C45	1.386 (3)	C18—C19	1.383 (3)
C45—H45	0.9500	C19—H19	0.9500
C45—C46	1.383 (3)	C19—C20	1.386 (3)
C46—H46	0.9500	C20—H20	0.9500
C31—C30	1.426 (3)	C5—C4	1.417 (3)
C31—C32	1.388 (3)	C5—C6	1.391 (3)
C30—C29	1.383 (3)	C4—C3	1.383 (3)
C29—H29	0.9500	C3—H3	0.9500
C29—C28	1.385 (3)	C3—C2	1.387 (3)
C28—H28	0.9500	C2—H2a	0.9500
C28—C27	1.380 (3)	C2—C1	1.375 (3)
C27—C32	1.394 (3)	C1—C6	1.394 (3)
C32—H32	0.9500	C6—H6	0.9500
C33—H33a	0.9800	C7—H7a	0.9800
C33—H33b	0.9800	C7—H7b	0.9800
C33—H33c	0.9800	C7—H7c	0.9800
			
C33—O6—C30	117.50 (15)	C7—O3—C4	117.24 (16)
C31—N4—C34	127.94 (17)	H2—N2—C8	112.6 (18)
H4—N4—C34	114.7 (18)	C5—N2—C8	122.36 (17)
H4—N4—C31	116.5 (18)	C5—N2—H2	114.2 (18)
O4—N3—O5	122.52 (18)	O1—N1—O2	122.52 (18)
C27—N3—O5	118.50 (16)	C1—N1—O2	118.72 (16)
C27—N3—O4	118.97 (16)	C1—N1—O1	118.76 (17)
C35—C34—N4	104.61 (15)	C9—C8—N2	106.31 (16)
C47—C34—N4	111.29 (16)	C21—C8—N2	110.58 (17)
C47—C34—C35	107.52 (15)	C21—C8—C9	105.36 (16)
C41—C34—N4	109.74 (15)	C15—C8—N2	108.77 (16)
C41—C34—C35	110.01 (16)	C15—C8—C9	111.68 (16)
C41—C34—C47	113.28 (16)	C15—C8—C21	113.87 (17)
C36—C35—C34	118.19 (18)	C10—C9—C8	121.87 (18)
C40—C35—C34	122.85 (18)	C14—C9—C8	119.73 (18)
C40—C35—C36	118.94 (19)	C14—C9—C10	118.31 (18)
H36—C36—C35	119.94 (12)	H10—C10—C9	119.57 (12)
C37—C36—C35	120.1 (2)	C11—C10—C9	120.9 (2)
C37—C36—H36	119.94 (13)	C11—C10—H10	119.57 (13)
H37—C37—C36	119.79 (13)	H11—C11—C10	119.82 (13)
C38—C37—C36	120.4 (2)	C12—C11—C10	120.4 (2)
C38—C37—H37	119.79 (13)	C12—C11—H11	119.82 (12)
H38—C38—C37	120.16 (13)	H12—C12—C11	120.20 (12)
C39—C38—C37	119.7 (2)	C13—C12—C11	119.61 (19)
C39—C38—H38	120.16 (13)	C13—C12—H12	120.20 (13)
H39—C39—C38	119.88 (13)	H13—C13—C12	120.00 (13)
C40—C39—C38	120.2 (2)	C14—C13—C12	120.0 (2)
C40—C39—H39	119.88 (13)	C14—C13—H13	120.00 (13)
C39—C40—C35	120.6 (2)	C13—C14—C9	120.8 (2)
H40—C40—C35	119.72 (12)	H14—C14—C9	119.60 (12)
H40—C40—C39	119.72 (13)	H14—C14—C13	119.60 (13)
C48—C47—C34	119.47 (17)	C22—C21—C8	119.17 (18)
C52—C47—C34	122.54 (17)	C26—C21—C8	122.3 (2)
C52—C47—C48	117.94 (19)	C26—C21—C22	118.4 (2)
H48—C48—C47	119.52 (12)	H22—C22—C21	119.45 (12)
C49—C48—C47	120.96 (19)	C23—C22—C21	121.1 (2)
C49—C48—H48	119.52 (12)	C23—C22—H22	119.45 (15)
H49—C49—C48	119.79 (12)	H23—C23—C22	120.13 (15)
C50—C49—C48	120.4 (2)	C24—C23—C22	119.7 (3)
C50—C49—H49	119.79 (13)	C24—C23—H23	120.13 (16)
H50—C50—C49	120.41 (13)	H24—C24—C23	120.05 (16)
C51—C50—C49	119.2 (2)	C25—C24—C23	119.9 (2)
C51—C50—H50	120.41 (13)	C25—C24—H24	120.05 (14)
H51—C51—C50	119.67 (13)	H25—C25—C24	119.81 (14)
C52—C51—C50	120.7 (2)	C26—C25—C24	120.4 (2)
C52—C51—H51	119.67 (12)	C26—C25—H25	119.81 (15)
C51—C52—C47	120.81 (19)	C25—C26—C21	120.5 (2)
H52—C52—C47	119.59 (12)	H26—C26—C21	119.77 (14)
H52—C52—C51	119.59 (12)	H26—C26—C25	119.77 (16)
C42—C41—C34	123.17 (18)	C16—C15—C8	123.83 (18)
C46—C41—C34	118.65 (18)	C20—C15—C8	117.86 (18)
C46—C41—C42	118.15 (18)	C20—C15—C16	118.14 (19)
H42—C42—C41	119.72 (12)	H16—C16—C15	119.54 (12)
C43—C42—C41	120.6 (2)	C17—C16—C15	120.9 (2)
C43—C42—H42	119.72 (13)	C17—C16—H16	119.54 (13)
H43—C43—C42	119.74 (13)	H17—C17—C16	119.84 (13)
C44—C43—C42	120.5 (2)	C18—C17—C16	120.3 (2)
C44—C43—H43	119.74 (13)	C18—C17—H17	119.84 (13)
H44—C44—C43	120.23 (13)	H18—C18—C17	120.32 (13)
C45—C44—C43	119.5 (2)	C19—C18—C17	119.4 (2)
C45—C44—H44	120.23 (13)	C19—C18—H18	120.32 (13)
H45—C45—C44	120.04 (13)	H19—C19—C18	119.75 (13)
C46—C45—C44	119.9 (2)	C20—C19—C18	120.5 (2)
C46—C45—H45	120.04 (13)	C20—C19—H19	119.75 (13)
C45—C46—C41	121.3 (2)	C19—C20—C15	120.7 (2)
H46—C46—C41	119.35 (12)	H20—C20—C15	119.65 (12)
H46—C46—C45	119.35 (13)	H20—C20—C19	119.65 (13)
C30—C31—N4	116.45 (18)	C4—C5—N2	117.61 (18)
C32—C31—N4	125.42 (18)	C6—C5—N2	124.45 (19)
C32—C31—C30	118.12 (17)	C6—C5—C4	117.85 (18)
C31—C30—O6	114.33 (16)	C5—C4—O3	113.88 (17)
C29—C30—O6	124.80 (17)	C3—C4—O3	125.07 (19)
C29—C30—C31	120.88 (18)	C3—C4—C5	121.0 (2)
H29—C29—C30	119.67 (12)	H3—C3—C4	119.59 (13)
C28—C29—C30	120.67 (18)	C2—C3—C4	120.8 (2)
C28—C29—H29	119.67 (11)	C2—C3—H3	119.59 (12)
H28—C28—C29	120.90 (11)	H2a—C2—C3	121.03 (12)
C27—C28—C29	118.21 (18)	C1—C2—C3	117.95 (19)
C27—C28—H28	120.90 (11)	C1—C2—H2a	121.03 (12)
C28—C27—N3	118.57 (17)	C2—C1—N1	119.50 (18)
C32—C27—N3	118.63 (17)	C6—C1—N1	117.66 (18)
C32—C27—C28	122.76 (18)	C6—C1—C2	122.8 (2)
C27—C32—C31	119.33 (18)	C1—C6—C5	119.5 (2)
H32—C32—C31	120.33 (11)	H6—C6—C5	120.26 (12)
H32—C32—C27	120.33 (12)	H6—C6—C1	120.26 (13)
H33a—C33—O6	109.5	H7a—C7—O3	109.5
H33b—C33—O6	109.5	H7b—C7—O3	109.5
H33b—C33—H33a	109.5	H7b—C7—H7a	109.5
H33c—C33—O6	109.5	H7c—C7—O3	109.5
H33c—C33—H33a	109.5	H7c—C7—H7a	109.5
H33c—C33—H33b	109.5	H7c—C7—H7b	109.5
			
O6—C30—C31—N4	−0.19 (19)	O3—C4—C5—N2	1.3 (2)
O6—C30—C31—C32	−179.47 (16)	O3—C4—C5—C6	177.91 (17)
O6—C30—C29—C28	−178.88 (19)	O3—C4—C3—C2	−178.2 (2)
O5—N3—C27—C28	−175.33 (19)	O2—N1—C1—C2	178.66 (18)
O5—N3—C27—C32	6.6 (2)	O2—N1—C1—C6	−2.2 (2)
O4—N3—C27—C28	5.6 (2)	O1—N1—C1—C2	−1.7 (2)
O4—N3—C27—C32	−172.43 (18)	O1—N1—C1—C6	177.38 (18)
N4—C34—C35—C36	−59.97 (18)	N2—C8—C9—C10	−139.86 (16)
N4—C34—C35—C40	121.49 (16)	N2—C8—C9—C14	43.7 (2)
N4—C34—C47—C48	162.14 (16)	N2—C8—C21—C22	−174.68 (16)
N4—C34—C47—C52	−15.09 (19)	N2—C8—C21—C26	1.6 (2)
N4—C34—C41—C42	142.93 (16)	N2—C8—C15—C16	−116.44 (17)
N4—C34—C41—C46	−39.12 (19)	N2—C8—C15—C20	58.65 (18)
N4—C31—C30—C29	179.90 (17)	N2—C5—C4—C3	−178.29 (18)
N4—C31—C32—C27	179.4 (2)	N2—C5—C6—C1	177.5 (2)
N3—C27—C28—C29	−176.91 (17)	N1—C1—C2—C3	178.96 (18)
N3—C27—C32—C31	178.54 (17)	N1—C1—C6—C5	−179.38 (17)
C34—N4—C31—C30	168.5 (2)	C8—N2—C5—C4	−157.32 (19)
C34—N4—C31—C32	−12.2 (2)	C8—N2—C5—C6	26.3 (2)
C34—C35—C36—C37	179.74 (19)	C8—C9—C10—C11	−178.86 (19)
C34—C35—C40—C39	179.20 (19)	C8—C9—C14—C13	178.52 (19)
C34—C47—C48—C49	−178.65 (18)	C8—C21—C22—C23	177.44 (19)
C34—C47—C52—C51	178.52 (18)	C8—C21—C26—C25	−177.7 (2)
C34—C41—C42—C43	178.11 (18)	C8—C15—C16—C17	173.58 (18)
C34—C41—C46—C45	−179.53 (17)	C8—C15—C20—C19	−175.86 (17)
C35—C34—N4—C31	−159.77 (15)	C9—C8—N2—C5	165.03 (15)
C35—C34—C47—C48	48.13 (18)	C9—C8—C21—C22	−60.21 (18)
C35—C34—C47—C52	−129.10 (15)	C9—C8—C21—C26	116.02 (17)
C35—C34—C41—C42	−102.49 (17)	C9—C8—C15—C16	126.54 (17)
C35—C34—C41—C46	75.46 (17)	C9—C8—C15—C20	−58.37 (18)
C35—C36—C37—C38	1.6 (3)	C9—C10—C11—C12	1.2 (2)
C35—C40—C39—C38	0.4 (2)	C9—C14—C13—C12	−0.4 (3)
C36—C35—C34—C47	58.44 (19)	C10—C9—C8—C21	102.7 (2)
C36—C35—C34—C41	−177.77 (19)	C10—C9—C8—C15	−21.4 (2)
C36—C35—C40—C39	0.7 (2)	C10—C9—C14—C13	1.9 (2)
C36—C37—C38—C39	−0.6 (3)	C10—C11—C12—C13	0.4 (2)
C37—C36—C35—C40	−1.7 (3)	C11—C10—C9—C14	−2.3 (2)
C37—C38—C39—C40	−0.4 (3)	C11—C12—C13—C14	−0.8 (2)
C40—C35—C34—C47	−120.1 (2)	C14—C9—C8—C21	−73.7 (2)
C40—C35—C34—C41	3.7 (2)	C14—C9—C8—C15	162.2 (2)
C47—C34—N4—C31	84.41 (18)	C21—C8—N2—C5	−81.10 (18)
C47—C34—C41—C42	17.87 (19)	C21—C8—C15—C16	7.4 (2)
C47—C34—C41—C46	−164.18 (16)	C21—C8—C15—C20	−177.56 (16)
C47—C48—C49—C50	0.4 (2)	C21—C22—C23—C24	−0.3 (3)
C47—C52—C51—C50	−0.3 (2)	C21—C26—C25—C24	1.1 (3)
C48—C47—C34—C41	−73.64 (19)	C22—C21—C8—C15	62.5 (2)
C48—C47—C52—C51	1.3 (2)	C22—C21—C26—C25	−1.4 (2)
C48—C49—C50—C51	0.5 (2)	C22—C23—C24—C25	0.0 (3)
C49—C48—C47—C52	−1.3 (2)	C23—C22—C21—C26	1.1 (3)
C49—C50—C51—C52	−0.6 (2)	C23—C24—C25—C26	−0.3 (3)
C52—C47—C34—C41	109.13 (19)	C26—C21—C8—C15	−121.3 (2)
C41—C34—N4—C31	−41.8 (2)	C15—C8—N2—C5	44.6 (2)
C41—C42—C43—C44	1.3 (2)	C15—C16—C17—C18	1.8 (2)
C41—C46—C45—C44	1.4 (2)	C15—C20—C19—C18	2.2 (2)
C42—C41—C46—C45	−1.5 (2)	C16—C15—C20—C19	−0.5 (2)
C42—C43—C44—C45	−1.4 (2)	C16—C17—C18—C19	0.0 (2)
C43—C42—C41—C46	0.2 (2)	C17—C16—C15—C20	−1.5 (2)
C43—C44—C45—C46	0.0 (2)	C17—C18—C19—C20	−2.0 (2)
C31—C30—O6—C33	175.28 (16)	C5—C4—O3—C7	179.77 (17)
C31—C30—C29—C28	1.0 (2)	C5—C4—C3—C2	1.3 (2)
C31—C32—C27—C28	0.6 (2)	C5—C6—C1—C2	−0.3 (2)
C30—C31—C32—C27	−1.4 (2)	C4—C5—C6—C1	1.2 (2)
C30—C29—C28—C27	−1.8 (2)	C4—C3—C2—C1	−0.4 (2)
C29—C30—O6—C33	−4.8 (2)	C3—C4—O3—C7	−0.7 (3)
C29—C30—C31—C32	0.6 (2)	C3—C4—C5—C6	−1.7 (2)
C29—C28—C27—C32	1.1 (2)	C3—C2—C1—C6	−0.1 (2)

### 2-Methoxy-N-(4-methylbenzyl)-5-nitroaniline (1) Crystal data

**Table:**

C_15_H_16_N_2_O_3_	*F*(000) = 576.366
*M**_r_* = 272.31	*D*_x_ = 1.347 Mg m^−^^3^
Monoclinic, *P*2_1_/*c*	Mo *K*α radiation, λ = 0.71073 Å
*a* = 9.5017 (4) Å	Cell parameters from 9923 reflections
*b* = 15.7855 (7) Å	θ = 2.5–27.6°
*c* = 8.9908 (4) Å	µ = 0.10 mm^−^^1^
β = 95.230 (2)°	*T* = 130 K
*V* = 1342.91 (10) Å^3^	Plate, orange
*Z* = 4	0.29 × 0.17 × 0.05 mm

### 2-Methoxy-N-(4-methylbenzyl)-5-nitroaniline (1) Data collection

**Table:**

Bruker APEXII CCD diffractometer	2497 reflections with *I*≥ 2u(*I*)
φ and ω scans	*R*_int_ = 0.050
Absorption correction: multi-scan (*SADABS*; Krause *et al.*, 2015)	θ_max_ = 27.5°, θ_min_ = 2.5°
*T*_min_ = 0.657, *T*_max_ = 0.746	*h* = −12→12
30573 measured reflections	*k* = −20→20
3078 independent reflections	*l* = −11→11

### 2-Methoxy-N-(4-methylbenzyl)-5-nitroaniline (1) Refinement

**Table:**

Refinement on *F*^2^	0 restraints
Least-squares matrix: full	0 constraints
*R*[*F*^2^ > 2σ(*F*^2^)] = 0.048	All H-atom parameters refined
*wR*(*F*^2^) = 0.136	*w* = 1/[σ^2^(*F*_o_^2^) + (0.0791*P*)^2^ + 0.4263*P*] where *P* = (*F*_o_^2^ + 2*F*_c_^2^)/3
*S* = 1.08	(Δ/σ)_max_ = 0.001
3078 reflections	Δρ_max_ = 0.33 e Å^−^^3^
245 parameters	Δρ_min_ = −0.38 e Å^−^^3^

### 2-Methoxy-N-(4-methylbenzyl)-5-nitroaniline (1) Fractional atomic coordinates and isotropic or equivalent isotropic displacement parameters (Å^2^)

**Table:**

	*x*	*y*	*z*	*U*_iso_*/*U*_eq_	
O3	0.76030 (11)	0.38217 (6)	0.01780 (12)	0.0242 (3)	
O2	0.97647 (13)	0.70533 (7)	−0.28049 (13)	0.0322 (3)	
O1	1.08131 (13)	0.61179 (8)	−0.40737 (13)	0.0365 (3)	
N1	1.00248 (13)	0.63104 (8)	−0.31186 (14)	0.0244 (3)	
N2	0.71393 (13)	0.54099 (8)	0.07966 (15)	0.0246 (3)	
C4	0.82050 (14)	0.43825 (9)	−0.07203 (15)	0.0197 (3)	
C9	0.63647 (15)	0.62833 (8)	0.28127 (16)	0.0214 (3)	
C5	0.79528 (14)	0.52434 (8)	−0.03534 (15)	0.0190 (3)	
C2	0.96011 (15)	0.48079 (9)	−0.26923 (16)	0.0222 (3)	
C1	0.93668 (14)	0.56407 (9)	−0.23134 (15)	0.0205 (3)	
C6	0.85415 (15)	0.58710 (9)	−0.11750 (15)	0.0207 (3)	
C10	0.50011 (16)	0.59885 (9)	0.29420 (17)	0.0251 (3)	
C14	0.71353 (15)	0.65970 (9)	0.40830 (17)	0.0240 (3)	
C3	0.89990 (15)	0.41751 (9)	−0.18848 (16)	0.0222 (3)	
C13	0.65483 (16)	0.66165 (9)	0.54449 (17)	0.0245 (3)	
C11	0.44275 (16)	0.60007 (10)	0.43046 (18)	0.0268 (3)	
C8	0.69961 (17)	0.62703 (9)	0.13353 (17)	0.0242 (3)	
C12	0.51903 (16)	0.63175 (9)	0.55794 (16)	0.0236 (3)	
C15	0.45916 (19)	0.63335 (11)	0.70754 (19)	0.0310 (4)	
C7	0.7786 (2)	0.29380 (9)	−0.0128 (2)	0.0321 (4)	
H3	0.913 (2)	0.3603 (13)	−0.211 (2)	0.032 (5)*	
H2a	1.0174 (18)	0.4693 (12)	−0.3504 (19)	0.025 (4)*	
H6	0.8408 (19)	0.6453 (13)	−0.094 (2)	0.029 (5)*	
H14	0.810 (2)	0.6791 (12)	0.403 (2)	0.028 (4)*	
H13	0.711 (2)	0.6824 (12)	0.632 (2)	0.028 (4)*	
H11	0.346 (2)	0.5786 (13)	0.437 (2)	0.037 (5)*	
H10	0.4443 (19)	0.5769 (12)	0.208 (2)	0.027 (4)*	
H8a	0.795 (2)	0.6540 (12)	0.1458 (19)	0.026 (4)*	
H8b	0.641 (2)	0.6586 (12)	0.057 (2)	0.028 (4)*	
H15a	0.505 (2)	0.5891 (14)	0.775 (2)	0.044 (6)*	
H15b	0.479 (2)	0.6901 (14)	0.758 (2)	0.044 (6)*	
H15c	0.359 (2)	0.6207 (13)	0.695 (2)	0.035 (5)*	
H7a	0.738 (2)	0.2788 (14)	−0.116 (2)	0.039 (5)*	
H7b	0.878 (2)	0.2780 (13)	0.000 (2)	0.039 (5)*	
H7c	0.729 (2)	0.2645 (14)	0.063 (2)	0.042 (5)*	
H2	0.698 (2)	0.5003 (14)	0.138 (2)	0.036 (5)*	

### 2-Methoxy-N-(4-methylbenzyl)-5-nitroaniline (1) Atomic displacement parameters (Å^2^)

**Table:**

	*U*^11^	*U*^22^	*U*^33^	*U*^12^	*U*^13^	*U*^23^
O3	0.0304 (6)	0.0152 (5)	0.0282 (5)	−0.0020 (4)	0.0089 (4)	−0.0017 (4)
O2	0.0386 (6)	0.0208 (5)	0.0386 (6)	−0.0037 (5)	0.0115 (5)	0.0021 (5)
O1	0.0446 (7)	0.0356 (6)	0.0323 (6)	−0.0083 (5)	0.0198 (5)	−0.0028 (5)
N1	0.0256 (6)	0.0254 (6)	0.0224 (6)	−0.0045 (5)	0.0034 (5)	0.0008 (5)
N2	0.0310 (7)	0.0147 (6)	0.0299 (6)	−0.0007 (5)	0.0130 (5)	−0.0006 (5)
C4	0.0193 (6)	0.0174 (6)	0.0220 (6)	−0.0013 (5)	0.0002 (5)	−0.0002 (5)
C9	0.0245 (7)	0.0132 (6)	0.0271 (7)	0.0028 (5)	0.0057 (5)	0.0004 (5)
C5	0.0182 (6)	0.0177 (6)	0.0211 (6)	−0.0009 (5)	0.0014 (5)	−0.0023 (5)
C2	0.0230 (7)	0.0242 (7)	0.0197 (6)	0.0003 (5)	0.0025 (5)	−0.0019 (5)
C1	0.0207 (6)	0.0213 (7)	0.0193 (6)	−0.0029 (5)	0.0011 (5)	0.0008 (5)
C6	0.0229 (7)	0.0169 (6)	0.0225 (7)	−0.0006 (5)	0.0024 (5)	−0.0006 (5)
C10	0.0236 (7)	0.0231 (7)	0.0284 (7)	0.0002 (5)	0.0013 (6)	−0.0038 (6)
C14	0.0223 (7)	0.0181 (6)	0.0320 (8)	−0.0011 (5)	0.0043 (6)	−0.0011 (5)
C3	0.0250 (7)	0.0183 (6)	0.0233 (7)	−0.0003 (5)	0.0021 (5)	−0.0034 (5)
C13	0.0275 (7)	0.0180 (6)	0.0277 (7)	0.0014 (5)	0.0004 (6)	−0.0019 (5)
C11	0.0211 (7)	0.0260 (7)	0.0338 (8)	−0.0008 (6)	0.0056 (6)	0.0002 (6)
C8	0.0306 (8)	0.0156 (6)	0.0276 (7)	0.0002 (5)	0.0083 (6)	−0.0010 (5)
C12	0.0272 (7)	0.0171 (6)	0.0274 (7)	0.0057 (5)	0.0066 (6)	0.0025 (5)
C15	0.0349 (9)	0.0296 (8)	0.0302 (8)	0.0042 (7)	0.0114 (7)	0.0036 (6)
C7	0.0456 (10)	0.0154 (7)	0.0367 (9)	−0.0030 (6)	0.0115 (7)	−0.0037 (6)

### 2-Methoxy-N-(4-methylbenzyl)-5-nitroaniline (1) Geometric parameters (Å, º)

**Table:**

O3—C4	1.3591 (16)	C10—C11	1.386 (2)
O3—C7	1.4354 (17)	C10—H10	0.962 (18)
O2—N1	1.2364 (17)	C14—C13	1.391 (2)
O1—N1	1.2280 (17)	C14—H14	0.969 (19)
N1—C1	1.4543 (17)	C3—H3	0.94 (2)
N2—C5	1.3716 (17)	C13—C12	1.389 (2)
N2—C8	1.4526 (17)	C13—H13	0.968 (19)
N2—H2	0.85 (2)	C11—C12	1.392 (2)
C4—C5	1.4238 (18)	C11—H11	0.99 (2)
C4—C3	1.3844 (19)	C8—H8a	1.000 (19)
C9—C10	1.391 (2)	C8—H8b	0.986 (19)
C9—C14	1.390 (2)	C12—C15	1.508 (2)
C9—C8	1.5067 (19)	C15—H15a	1.00 (2)
C5—C6	1.3841 (19)	C15—H15b	1.01 (2)
C2—C1	1.3811 (19)	C15—H15c	0.96 (2)
C2—C3	1.388 (2)	C7—H7a	1.00 (2)
C2—H2a	0.967 (17)	C7—H7b	0.98 (2)
C1—C6	1.3931 (19)	C7—H7c	0.98 (2)
C6—H6	0.95 (2)		
			
C7—O3—C4	117.02 (11)	C2—C3—C4	120.28 (13)
O1—N1—O2	122.79 (12)	H3—C3—C4	118.9 (12)
C1—N1—O2	118.16 (12)	H3—C3—C2	120.8 (12)
C1—N1—O1	119.05 (12)	C12—C13—C14	121.27 (14)
C8—N2—C5	120.46 (12)	H13—C13—C14	119.2 (11)
H2—N2—C5	117.8 (13)	H13—C13—C12	119.5 (11)
H2—N2—C8	118.0 (13)	C12—C11—C10	121.05 (14)
C5—C4—O3	113.27 (12)	H11—C11—C10	119.7 (11)
C3—C4—O3	125.68 (12)	H11—C11—C12	119.3 (11)
C3—C4—C5	121.05 (12)	C9—C8—N2	111.32 (11)
C14—C9—C10	118.37 (13)	H8a—C8—N2	108.9 (10)
C8—C9—C10	121.18 (13)	H8a—C8—C9	109.1 (10)
C8—C9—C14	120.45 (13)	H8b—C8—N2	107.7 (11)
C4—C5—N2	118.42 (12)	H8b—C8—C9	111.4 (11)
C6—C5—N2	123.24 (12)	H8b—C8—H8a	108.4 (14)
C6—C5—C4	118.34 (12)	C11—C12—C13	117.92 (14)
C3—C2—C1	118.19 (13)	C15—C12—C13	120.06 (14)
H2a—C2—C1	118.6 (11)	C15—C12—C11	122.01 (14)
H2a—C2—C3	123.2 (11)	H15a—C15—C12	110.5 (12)
C2—C1—N1	118.87 (12)	H15b—C15—C12	110.1 (12)
C6—C1—N1	118.18 (12)	H15b—C15—H15a	107.2 (17)
C6—C1—C2	122.94 (13)	H15c—C15—C12	110.0 (12)
C1—C6—C5	119.16 (13)	H15c—C15—H15a	106.9 (17)
H6—C6—C5	120.2 (11)	H15c—C15—H15b	112.0 (17)
H6—C6—C1	120.6 (11)	H7a—C7—O3	111.3 (12)
C11—C10—C9	120.89 (14)	H7b—C7—O3	111.1 (13)
H10—C10—C9	120.2 (10)	H7b—C7—H7a	109.7 (17)
H10—C10—C11	118.9 (10)	H7c—C7—O3	104.4 (13)
C13—C14—C9	120.49 (13)	H7c—C7—H7a	111.3 (17)
H14—C14—C9	120.3 (11)	H7c—C7—H7b	108.8 (17)
H14—C14—C13	119.2 (11)		
			
O3—C4—C5—N2	−1.32 (14)	C9—C14—C13—C12	0.66 (16)
O3—C4—C5—C6	178.65 (11)	C9—C8—N2—C5	−166.23 (12)
O3—C4—C3—C2	−177.79 (14)	C5—C4—O3—C7	179.06 (13)
O2—N1—C1—C2	177.42 (13)	C5—C4—C3—C2	1.88 (16)
O2—N1—C1—C6	−3.49 (15)	C5—C6—C1—C2	1.67 (16)
O1—N1—C1—C2	−2.74 (15)	C6—C5—N2—C8	−7.66 (17)
O1—N1—C1—C6	176.35 (13)	C6—C5—C4—C3	−1.06 (15)
N1—C1—C2—C3	178.17 (13)	C6—C1—C2—C3	−0.87 (16)
N1—C1—C6—C5	−177.38 (12)	C10—C9—C14—C13	−0.23 (15)
N2—C5—C4—C3	178.97 (13)	C10—C11—C12—C13	−0.38 (17)
N2—C5—C6—C1	179.29 (14)	C10—C11—C12—C15	−179.69 (14)
N2—C8—C9—C10	−63.40 (15)	C14—C9—C10—C11	−0.48 (15)
N2—C8—C9—C14	116.98 (13)	C14—C13—C12—C11	−0.34 (16)
C4—C5—N2—C8	172.31 (13)	C14—C13—C12—C15	178.98 (13)
C4—C5—C6—C1	−0.68 (15)	C3—C4—O3—C7	−1.25 (18)
C4—C3—C2—C1	−0.92 (16)	C13—C14—C9—C8	179.40 (13)
C9—C10—C11—C12	0.80 (17)	C11—C10—C9—C8	179.88 (13)
